# Author Correction: The trans-kingdom battle between donor and recipient gut microbiome influences fecal microbiota transplantation outcome

**DOI:** 10.1038/s41598-021-82644-z

**Published:** 2021-02-19

**Authors:** Negin Kazemian, Milad Ramezankhani, Aarushi Sehgal, Faizan Muhammad Khalid, Amir Hossein Zeinali Kalkhoran, Apurva Narayan, Gane Ka‑Shu Wong, Dina Kao, Sepideh Pakpour

**Affiliations:** 1 School of Engineering, University of British Columbia, Kelowna, BC Canada; 2 Department of Computer Science and Engineering, National Institute of Technology, Hamirpur, Himachal Pradesh India; 3 Department of Computer Science, University of British Columbia, Kelowna, BC Canada; 4 Department of Biological Sciences, University of Alberta, Edmonton, AB Canada; 5 Department of Medicine, University of Alberta, Edmonton, AB Canada; 6 BGI-Shenzhen, Beishan Industrial Zone, Yantian District, Shenzhen, China; 7 Division of Gastroenterology, Department of Medicine, University of Alberta, Edmonton, AB Canada

Correction to: *Scientific Reports* https://doi.org/10.1038/s41598-020-75162-x, published online 27 october 2020

The original version of this Article contained errors.

Information on the Data Availability was incomplete. Therefore, the original Data Availability text,

“The normalized and non-normalized feature tables are available in supplementary data”.

Now reads,

“The normalized and non-normalized feature tables are available in supplementary data. The raw metagenomics sequence data is available at https://www.ncbi.nlm.nih.gov/bioproject/PRJNA684000”.

This has now been corrected in the PDF and HTML versions of the Article.

