# Supplementary material for: The trans-kingdom battle between donor and recipient gut microbiome influences fecal microbiota transplantation outcome
Source: Sci Rep. 2020 Oct 27;10:18349. doi: 10.1038/s41598-020-75162-x (PMC7591866; doi:10.1038/s41598-020-75162-x)
Supplement: Supplementary file 2 — Supplementary Information 2. [file 41598_2020_75162_MOESM2_ESM.pdf]

Supplementary Information

A trans-kingdom battle between donor and recipient gut microbiome influences fecal microbiota transplantation outcome

Negin Kazemian[1], Milad Ramezankhani[1], Aarushi Sehgal[2], Faizan Muhammad Khalid[1], Amir Hossein Zeinali Kalkhoran[3], Apurva Narayan[3], Gane Ka-Shu Wong[4,5,6,] Dina Kao[7*] & Sepideh Pakpour[1*]

[1]*School of Engineering, University of British Columbia, Kelowna, BC, Canada*
[2]*Department of Computer Science and Engineering, National Institute of Technology, Hamirpur, Himachal Pradesh, India*
[3]*Department of Computer Science, University of British Columbia, Kelowna, BC, Canada*
[4]*Department of Biological Sciences, University of Alberta, Edmonton, Alberta, Canada*
[5]*Department of Medicine, University of Alberta, Edmonton, Alberta, Canada*
[6]*BGI-Shenzhen, Beishan Industrial Zone, Yantian District, Shenzhen, China*
[7]*Division of Gastroenterology, Department of Medicine, University of Alberta, Edmonton, Alberta, Canada*

* Corresponding authors
sepideh.pakpour@ubc.ca (SP)
dkao@ualberta.ca (DK)

Table S1. Clinical characteristics of rCDI patients receiving FMT. Among recipients, 9/17 patients were successfully treated with a single FMT (53% successful FMT), while 8/17 patients failed the first FMT and required a second procedure. There was no difference between the two groups in factors of age (p=0.7039), sex (p=1.000), or duration of rCDI pre-FMT (p=0.4237). The Shapiro-Wilk test was used to determine if data were normally distributed. If normally distributed, a Student t-test was used, and if not the nonparametric Mann–Whitney U test was performed.

	**Single FMT**	**Repeated FMT**
**Number of patients**	9	8
**Age**	67.7 (35.3-84.9)	71.4 (49.5-83.8)
**Gender**	6 male; 3 female	5 male; 3 female
**Average duration of rCDI pre-FMT**	150.4 days (71-275)	126.2 days (53-246)

Numbers are given as means with range in brackets.

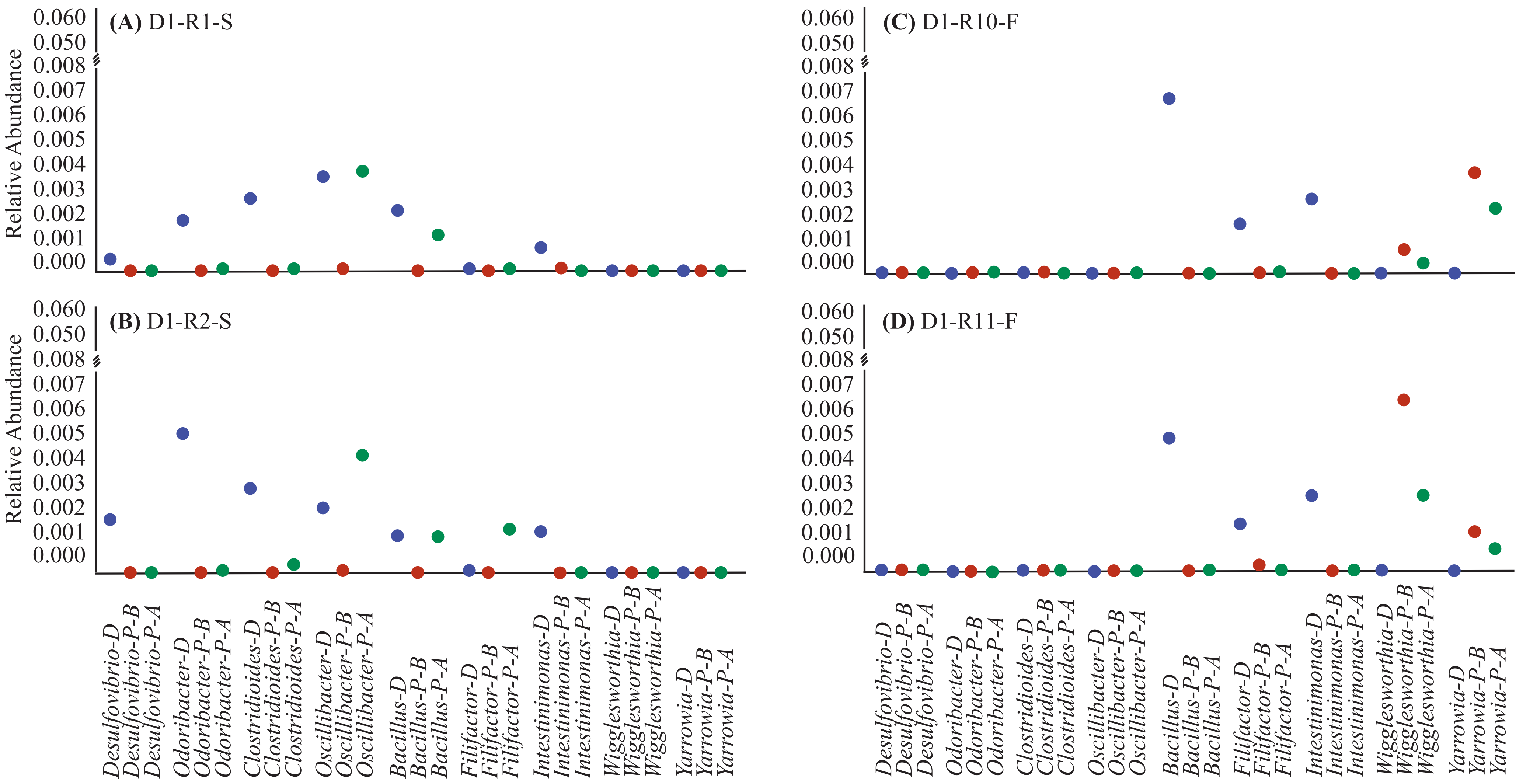

Fig. S1 The intra-variability of relative abundance of the top 9 features of donor 1 (**D1**) samples and their corresponding recipients, pre- and post-FMT leading to successful (**A** and **B**) and failed (**C** and **D**) FMT outcomes.

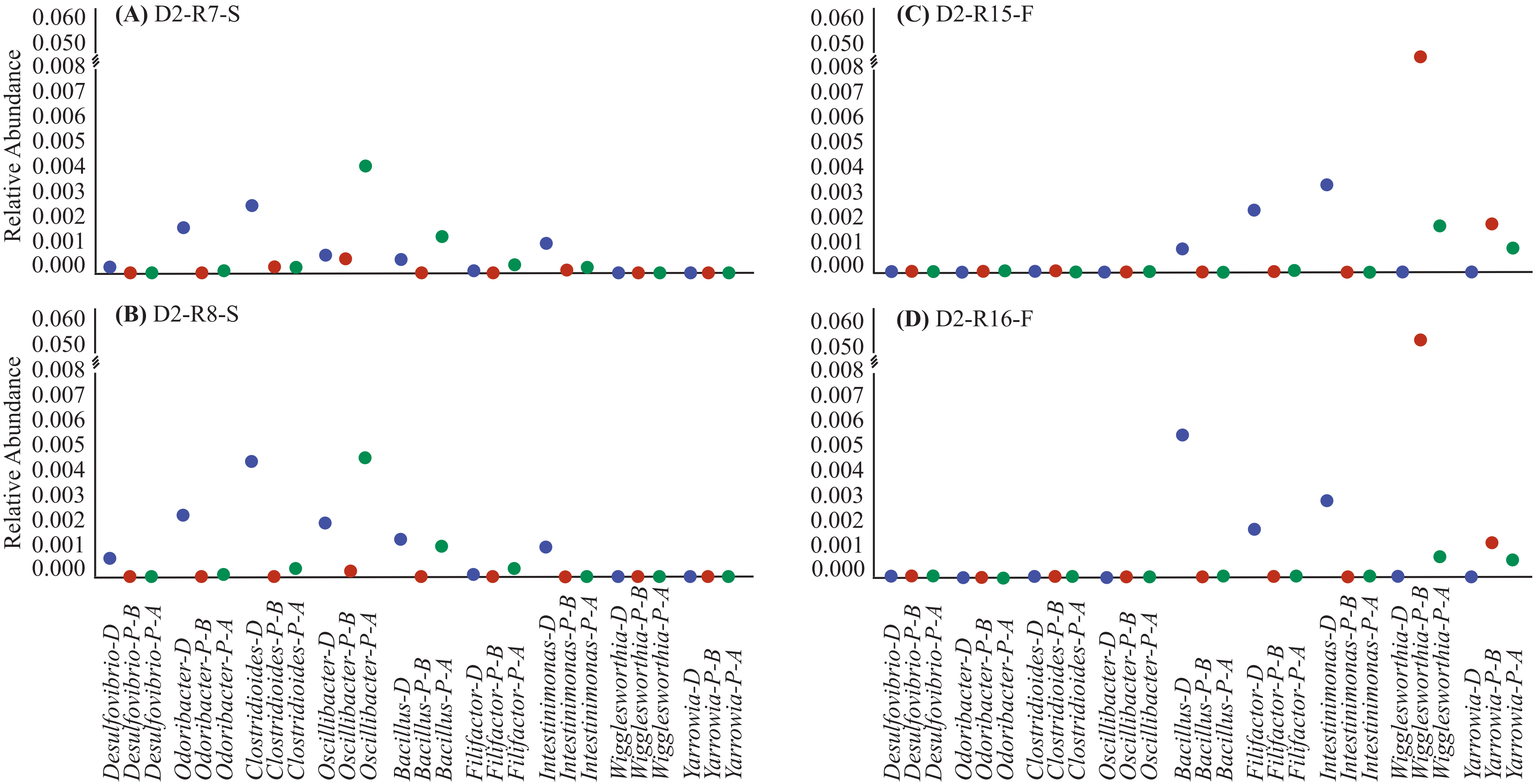

Fig. S2 The intra-variability of relative abundance of the top 9 features of donor 3 (**D3**) samples and their corresponding recipients, pre- and post-FMT leading to successful (**A** and **B**) and failed (**C** and **D**) FMT outcomes.
